# FGF1 improves functional recovery through inducing PRDX1 to regulate autophagy and anti‐ROS after spinal cord injury

**DOI:** 10.1111/jcmm.13566

**Published:** 2018-03-07

**Authors:** Jiawei Li, Qingqing Wang, Hanxiao Cai, Zili He, Haoli Wang, Jian Chen, Zengming Zheng, Jiayu Yin, Zhiyong Liao, Huazi Xu, Jian Xiao, Fanghua Gong

**Affiliations:** ^1^ The Second Affiliated Hospital and Yuying Children's Hospital of Wenzhou Medical University Wenzhou Zhejiang China; ^2^ School of Pharmacy Wenzhou Medical University Wenzhou Zhejiang China; ^3^ School of Life and Environmental Science Wenzhou University Wenzhou Zhejiang China

**Keywords:** autophagy, fibroblast growth factor 1, functional recovery, spinal cord injury

## Abstract

Fibroblast growth factor 1 (FGF1) is thought to exert protective and regenerative effects on neurons following spinal cord injury (SCI), although the mechanism of these effects is not well understood. The use of FGF1 as a therapeutic agent is limited by its lack of physicochemical stability and its limited capacity to cross the blood‐spinal cord barrier. Here, we demonstrated that overexpression of FGF1 in spinal cord following SCI significantly reduced tissue loss, protected neurons in the ventricornu, ameliorated pathological morphology of the lesion, dramatically improved tissue recovery via neuroprotection, and promoted axonal regeneration and remyelination both in vivo and in vivo. In addition, the autophagy and the expression levels of PRDX1 (an antioxidant protein) were induced by AAV‐FGF1 in PC12 cells after H_2_O_2_ treatment. Furthermore, the autophagy levels were not changed in PRDX1‐suppressing cells that were treated by AAV‐FGF1. Taken together, these results suggest that FGF1 improves functional recovery mainly through inducing PRDX1 expression to increase autophagy and anti‐ROS activity after SCI.

## 1 INTRODUCTION

Traumatic spinal cord injury (SCI) is a significant contributor to many neurological complications, including paraplegia or quadriplegia. These debilitating situations affect morbidity, and SCI treatment is limited.^1^, ^2^ SCI is classified into primary and secondary stages.^3^ The primary stage includes mechanical impaction to spinal cord, such as compression, stretching or contusion.^4^ Secondary injury is considered a multi‐factorial and complicated stage that starts after primary SCI and can last weeks or even months.^5^ Hypoxia‐ischaemia (HI) at the lesion site of the spinal cord, resulting from primary mechanical trauma, affects the production of active oxygen and free radicals (reactive oxygen species, ROS),^6^ one of the major detrimental effects during secondary injury.^7^, ^8^ Autophagy is an intracellular degradation pathway that delivers cytoplasmic components to the lysosome, and is involved in many essential physiological processes, such as axon formation.^9^, ^10^ However, the dysregulation of autophagy may contribute to numerous pathological conditions.^11^ One type of autophagy, chaperone‐mediated autophagy (CMA), was reported to be triggered by resisting ROS‐induced motoneuronal death during spinal cord destruction.^12^, ^13^ These results suggest that autophagy may play an important role in acute trauma.

PRDX1 is expressed mainly in the cytosol and participates in the regulation of several ROS‐dependent signalling pathways and is considered a key intracellular factor maintaining the balance of cell survival and apoptosis.^14^, ^15^ Most studies of PRDX1 have focused on its effect in cancer, but there have been only few investigating its role in acute injury. Fibroblast growth factors (FGFs) are expressed in several systems during cell generation, differentiation and migration.^16^ Fibroblast growth factor 1 (FGF1) plays a crucial role in neuroprotection and axon regeneration in the nervous system, and its use has been examined for safety and feasibility by clinical trials.^17^ However, administration of FGF1 by either subcutaneous or intravenous is ineffective in the treatment of SCI because FGF1 is a macromolecular protein with poor penetrability of the blood‐spinal cord barrier (BSCB). Therefore, it is essential to find an effective delivery route for FGF1 with sustained release. Adeno‐associated virus (AAV) can be used to remarkably promote gene expression with efficiency and longevity.^18^ Here, the results showed that PRDX1 and autophagy are both augmented after up‐regulation of FGF1, which indicate that FGF1 can influence ROS and autophagy to promote functional recovery after SCI. Additionally, PRDX1 may play a key role in mediation of the interaction between ROS and autophagy.

## 2 MATERIALS AND METHODS

### 2.1 Usage of animals and ethics statement

Adult female Sprague Dawley rats (200‐220 g, n = 80) were acquired from the Animal Center of the Chinese Academy of Science (Shanghai, China). The care and use of all animals conformed to guidelines set forth by the Chinese National Institutes of Health. The rats were housed under strictly controlled environmental conditions.

### 2.2 AAV vector construction and virus production

The AAV‐2 particles of FGF1 and lentivirus particles of PRDX1 were constructed and synthesized as previous study described.^19^ Spinal cord microinjection of the recombinant AAV vector was performed with a stereotaxic instrument.

### 2.3 Cell culture and virus transfection

PC12 cells were obtained from the Cell Storage Center of Wuhan University (Wuhan, China). Cells were maintained under appropriate conditions for proliferation using RMPI 1640 containing heat‐inactivated 10% FBS, and were cultured in a humidified atmosphere of 5% CO_2_ and 95% air at 37°C. AAV‐FGF1 (1 × 10^9^ TU) and LV‐PRDX1 (MOI = 20) were added to PC12 cells at 24 hours after plating for 12 hours.

To study the effect of FGF1 on autophagy of PC12 cells, we treated the cells with 3‐methyladenine (3‐MA) (10 mmol/L; 2 hours) or chloroquine (CQ; 50 μmol/L; 2 hours) at 24 hours after virus transfection.

### 2.4 Spinal cord injury model and virus injection

Spinal cord injury protocols were carried out in adult female SD rats as previously described.^20^ Immediately after SCI, 10 μL of AAV was orthotopically injected at a dose of 1 × 10^9^ TU using a microsyringe. The muscle and skin were sutured layer by layer and then sterilized by iodine. Post‐operative monitoring included manual bladder emptying 3 times each day. Subsequently, the animals were killed at 7 or 14 days after SCI.

### 2.5 Locomotion recovery assessment

Locomotion recovery was assessed using the Basso‐Beattie‐Bresnahan (BBB) locomotion scale and footprint analysis at 0, 1, 3, 5, 7 and 14 days. Rats were placed in an open experimental field and allowed to move freely for 5 minutes. Crawling ability was assessed by the BBB scale ranging from 0 (no limb movement or weight support) to 21 (normal locomotion). The footprint analysis was performed by dipping the animal's posterior limb with red dye and the forelimb with blue dye. Evaluations were performed by 5 independent examiners who were blinded to the experimental conditions.

### 2.6 Tissue preparation

At specific time‐points following SCI, the rats were anaesthetized with 10% chloral hydrate (ip, 3.5 mL/kg) and perfused with 0.9% saline solution via cardiac puncture. For H&E, Luxol fast blue (LFB) and immunohistochemistry stain, animals were perfused with 4% paraformaldehyde in 0.1 mol/L phosphate‐buffered saline (PBS) after saline solution. Centred at the lesion site, 0.5‐cm section of the spinal cord was separated, post‐fixed by 4% paraformaldehyde for 6 hours and then embedded in paraffin. Longitudinal or transverse sections (5 μm thick) were mounted on slides for subsequent staining. For Western blot, a spinal cord segment (0.5 cm length) at the contusion epicentre was dissected and immediately stored at −80°C.

### 2.7 Western blot

For in vivo protein analysis, a spinal cord segment at the lesion epicentre was dissected at 7 and 14 days and immediately stored at −80°C. Proteins from animal tissue or PC12 cells were first quantified by the BCA reagent method. We separated the proteins on 8% (w/v) or 12% (w/v) gels and transferred the proteins onto polyvinylidene fluoride membrane (Bio‐Rad, Hercules, CA, USA). Membranes were blocked with 5% (w/v) milk (Bio‐Rad) in TBS with 0.05% Tween 20 (TBST) for 2 hours at room temperature and then were incubated overnight at 4°C with primary antibodies of FGF1 (1:1000; Abcam), ace‐tubulin (1:2000; Cell Signaling Technology), Tau (1:1000; Abcam), GAP43 (1:1000; Abcam), MBP (1:1000; Abcam), p‐mTOR (1:1000; Cell Signaling Technology), mTOR (1:1000; Cell Signaling Technology), ATG7 (1:1000; Bio‐world), Beclin1 (1:1000; Abcam), LC‐3 I/II (1:1000; Cell Signaling Technology), PRDX1 (1:1000; Cell Signaling Technology) and GAPDH (1:10000; Bio‐world). Next, the membranes were washed with TBST 3 times and treated with horseradish peroxidase‐conjugated secondary antibodies for 60 minutes. All signals were detected by ChemiDocXRS + Imaging System (Bio‐Rad). All experiments were repeated 3 times for accuracy.

### 2.8 Histology and immunofluorescence staining

Longitudinal or transverse sections mounted on slides were prepared as previously described.^21^ The sections for histopathological examination were subjected to H&E staining and LFB staining according to manufacturer's instructions.

For immunofluorescence staining, sections were processed with primary antibodies against the following proteins: GFAP (1:1000; Santa Cruz), ace‐tubulin (1:1000; Cell Signaling Technology), NeuN (1:500; Abcam), GAP43 (1:500; Abcam) and LC3 (1:500; Cell Signaling Technology). The sections were washed with PBST 4 times for 3 minutes each and then incubated with Alexa Fluor 568, Alexa Fluor 488 or Alexa Fluor 647 donkey anti‐rabbit/antimouse secondary antibodies for 1 hour at 37°C. Next, the sections were washed with PBST 3 times, incubated with DAPI for 7 minutes, washed with PBST and then sealed with a coverslip. The images were captured by a confocal fluorescence microscope (Nikon, Japan).

### 2.9 Measurement of intracellular ROS levels

PC12 cells were plated in 6‐well plates (2.0 × 10^5^ cells/well), then allowed to attach for 24 hours and then transformed with AAV vector for 12 hours. Next, the cells were exposed to the H_2_O_2_ condition for 8 hours and then stained the cells with 10 mmol/L DCFH‐DA for 30 minutes at 37°C. ROS levels were evaluated by observation by a confocal fluorescence microscope in cells stained with DCFH‐DA, and the strength of fluorescence was measured and subjected to statistical analysis.

### 2.10 Statistical analysis

All data were expressed as means ± SEM. Differences between groups in BBB scales were analysed with 1‐way ANOVA followed by Tukey's multiple comparison test. Statistical analysis of the other data was performed with 1‐way analysis of variance (ANOVA). All statistical analyses were conducted using GraphPad Prism 5 for Windows. Differences were considered statistically significant when *P *<* *.05.

## 3 RESULTS

### 3.1 Feasibility of AAV vector to mediate the overexpression of FGF1 for SCI treatment

**Figure 1:**
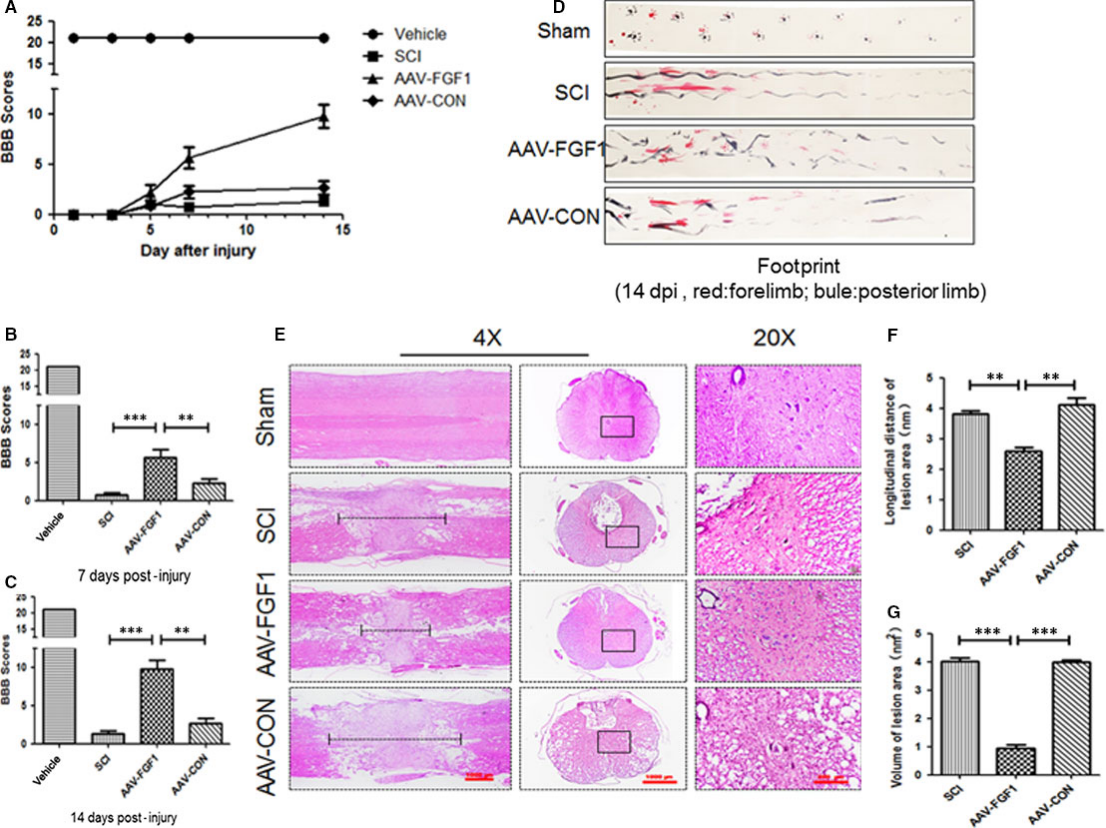
FGF1 mediated by AAV vector promoted motor functional recovery after spinal cord injury (SCI). A, The Basso‐Beattie‐Bresnahan (BBB) locomotion scales of the sham, SCI, AAV‐FGF1 and AAV‐CON groups (n = 10 per group) at 1, 3, 5, 7 and 14 days after SCI. B and C, Quantification of BBB locomotion scales at 7 and 14 days from A. ***P <* *.001; **P <* *.05. D, Footprint analysis results of the different groups. E, Representative images from H&E at 14 dpi. Scale bar = 1000 μm (4×), Scale bar = 200 μm (20×). F, Quantification of longitudinal distance of lesion area (nm) from D, **P <* *.01. G, Quantification of the volume of the lesion area (nm^2^) from D, ***P <* *.001

In this study, AAV‐FGF1 (5 × 10^11^ pfu/kg) was injected into the lesion area of rat immediately after SCI. As shown in Figure 1A, the sham‐operated group had an average score of 21, representing normal motor function. There was no significant difference between the groups (SCI group, the SCI + AAV‐FGF1 group and the SCI + AAV‐CON group) in the early stage (1, 3 and 5 dpi). However, with a longer time of observation, SCI + AAV‐FGF1 group showed better posterior limb motor function and scored twofold to threefold higher than rats in the SCI group and the SCI + AAV‐CON group at 7 dpi (days post‐injury) and 14 dpi (Figure 1A‐C). Furthermore, footprint analysis revealed differences in the track of the posterior limb. The track of animals from the AAV‐FGF1 group showed inconsistent motor function and extensive dragging, appearing as a wavy line in the SCI and SCI + AAV‐CON groups (Figure 1D), but animals in the SCI + AAV‐FGF1 group showed fairly consistent posterior limb co‐ordination with little stumbling at 14 dpi (shown in blue in Figure 1D). Additionally, the H&E staining was performed to examine the histological morphology through observing longitudinal and transverse sections. Compared to the sham group, there was obvious devastating damage in the central grey matter and the dorsal white matter. Consistent with the locomotion evaluation, the AAV‐FGF1–treated group showed less damage of the tissue and neurons, with a smaller lesion area (Figure 1E‐G), indicating that the increased FGF1 provided by the AAV vector reduced tissue loss, protected neurons in the ventricornu and ameliorated the pathological morphology of the lesion area.

### 3.2 AAV‐FGF1 promoted neuroprotection, axon regeneration and remyelination after SCI

**Figure 2:**
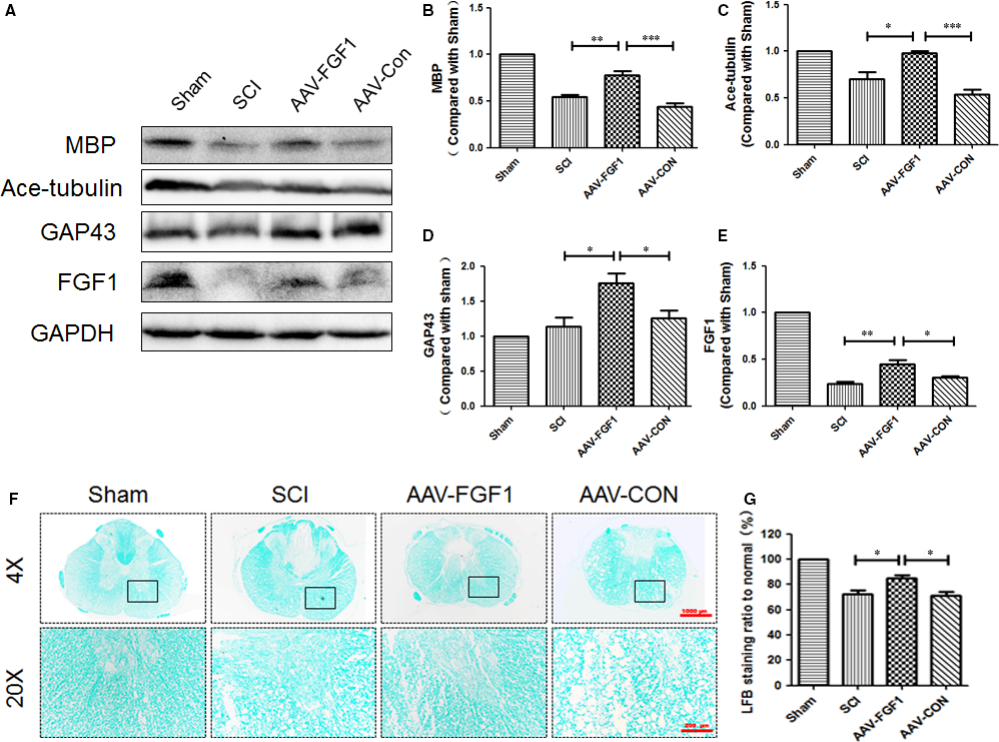
AAV‐FGF1 promoted neuroprotection, axon regeneration and remyelination after spinal cord injury. A, Representative Western blot result of MBP, ace‐tubulin, GAP43 and FGF1 at 14 dpi in each group. B‐E, Quantification of Western blot data from A, ***P <* *.001; **P <* *.01; *P <* *.05. F, Representative images of white matter with LFB staining and transmission electron microscopy images of the myelin sheath at 14 dpi. Scale bar = 1000 μm (4×), Scale bar = 200 μm (20×). G, Quantification of LFB staining ratio to normal (%) from F, all data represent mean values ± SEM, n = 5, *P <* *.05

**Figure 3:**
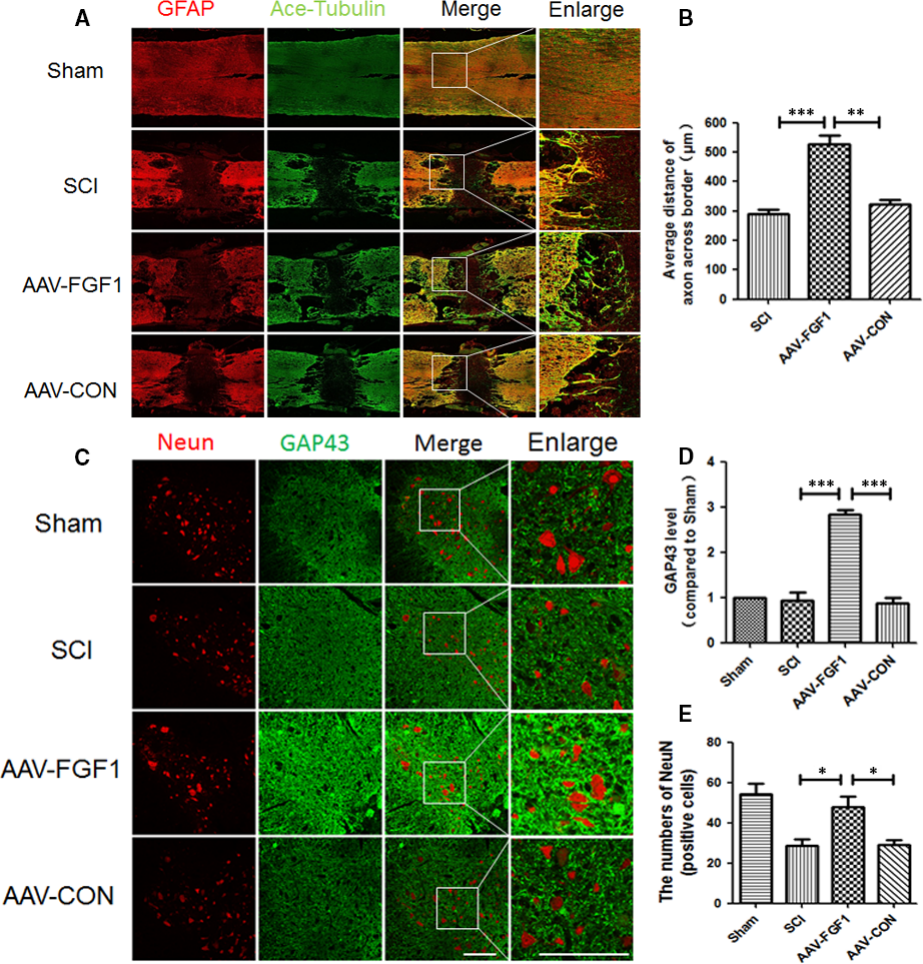
AAV‐FGF1 enhanced axon migration and protected neurons. A, Co‐immunofluorescence images show GFAP (red) and ace‐tubulin (green) after spinal cord injury (SCI) in each group. Scale bar = 1000 μm and Scale bar = 200 μm (enlarged region). B, Quantification of fluorescence (average distance of axons across border (μm)) from A, ***P <* *.001; **P <* *.01. C, Co‐immunofluorescence images show NeuN (red) and GAP43 (green) after SCI in each group. Scale bar = 200 μm. D, Quantification of fluorescence (GAP43 level) from C, ***P <* *.001. E, Quantification of fluorescence (numbers of NeuN) from C, *P <* *.05

To determine the effect of AAV‐FGF1 on SCI, the expression levels of MBP (myelin basic protein), acetylated‐tubulin (ace‐tubulin; stabilized microtubules in axon) and GAP43 (neuronal protectional protein) were measured in each group at 14 days after SCI. As shown in Figure 2A‐D, levels of MBP, ace‐tubulin and GAP43 were significantly increased for the AAV‐FGF1 group compared to those of the SCI and SCI + AAV‐FGF1 groups. The results of LFB staining showed that the LFB‐positive myelin tissues in the SCI + AAV‐FGF1 group showed denser white matter than those in either the SCI or the SCI + AAV‐CON group (Figure 2F‐G) at 14 dpi. FGF1 levels were also examined in vivo at 14 dpi, the FGF1 protein levels were significantly increased in AAV‐FGF1–treated rat (Figure 2A,E). Additionally, co‐immunofluorescence of ace‐tubulin, indicating axons in the process of regeneration, and GFAP, indicating astrocytes, was performed at 14 dpi, and longitudinal sections were examined to assess axon regeneration (Figure 3A). The GFAP‐positive astrocytes were distributed along the lesion border. Notably, in the SCI + AAV‐FGF1 group, there were more ace‐tubulin–positive, injured axons that crossed the lesion border and extended farther from the rostral border of the lesion site compared with the axons observed in the SCI and SCI + AAV‐CON groups. To verify the neuronal protection conferred by AAV‐FGF1, co‐immunostaining of NeuN (a marker of neurons) and GAP43 was performed for transverse sections, and the ventral motor neurons (VMNs) were counted (Figure 3B,C). The results showed that AAV‐FGF1 remarkably reduced the loss of VMNs and increased the expression of GAP43. Taken together, up‐regulation of FGF1 by AAV vector dramatically improved tissue recovery by promotion of neuroprotection, axon regeneration and remyelination after SCI.

### 3.3 FGF1 increased autophagy after SCI

**Figure 4:**
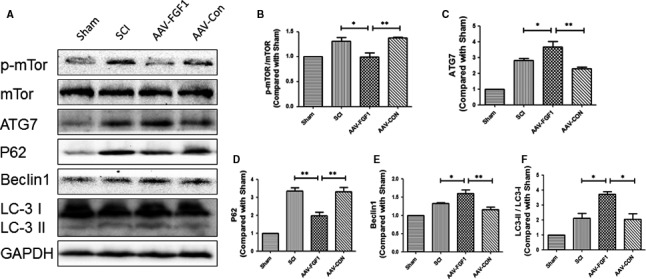
FGF1 increased autophagy after spinal cord injury. A, Representative Western blot results of autophagy‐associated proteins in each group at 7 dpi. B‐F, Quantification of Western blot data from A, **P <* *.01; *P <* *.05

As previously described, autophagy plays a crucial role in the pathology and the restoration of SCI. To determine the impact of FGF1 on the degree of autophagy, the levels of proteins involved in the autophagy signalling pathway were measured, including mTOR, ATG7, P62, Beclin1 and LC3 at 7 dpi (Figure 4A‐F). The results showed that phosphorylation of mTOR was observably decreased after treatment with AAV‐FGF1 following SCI. In contrast, the levels of ATG7, Beclin1 and LC3 II/I were significantly increased in the SCI + AAV‐FGF1 group compared with the levels in the injured group and the control vector group. The expression level of P62, a biomarker of mature autophagic vesicles, was markedly decreased in the SCI + AAV‐FGF1 group compared with that of the injured group and the control group. Overall, these data suggest that administration of FGF1 could enhance the expression of autophagy‐associated proteins to stimulate the level of autophagy after injury. It seems reasonable that this effect may be related to the therapeutic effect of FGF1.

### 3.4 FGF1 protects the cytoskeleton and promotes cell polarization in vitro

**Figure 5:**
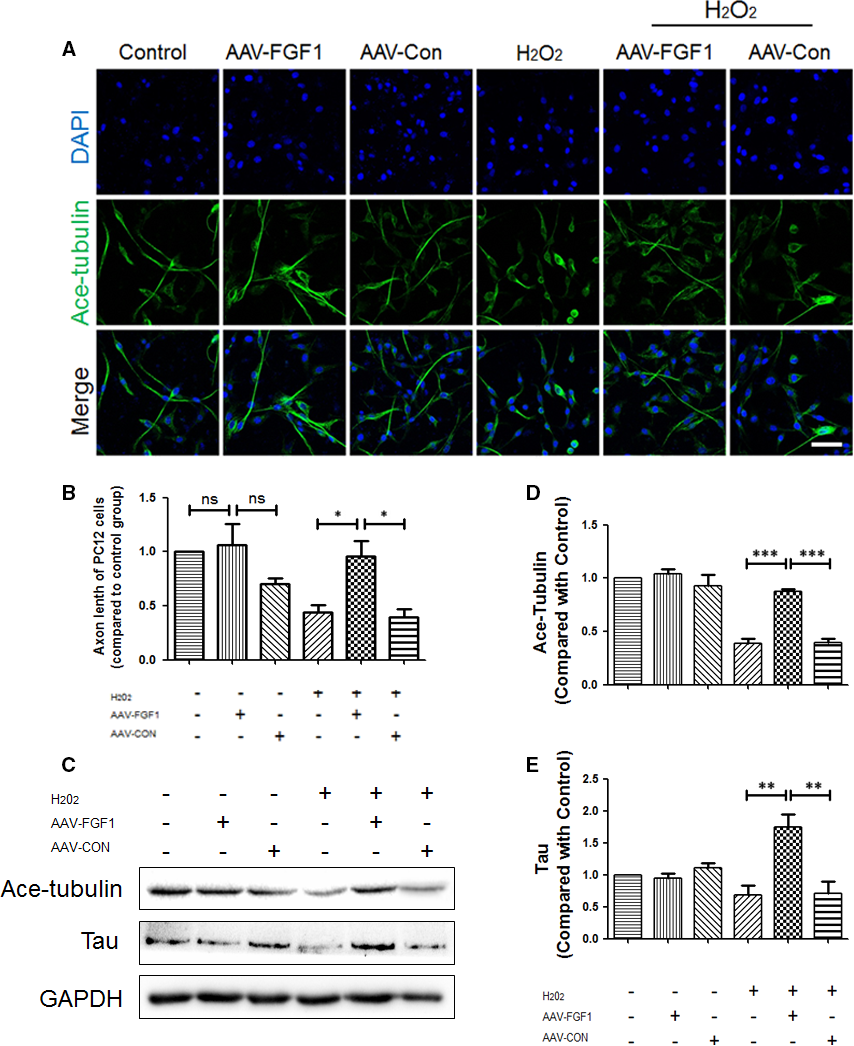
FGF1 protects the cytoskeleton and promotes cell polarization in vitro. A, Immunofluorescence images show ace‐tubulin (green) in PC12 cells subjected to H_2_O_2_ (100 μmol/L) and treated with AAV‐FGF1. Scale bar = 50 μm. B, Quantification of fluorescence (axonal length of PC12 cells) from A, ns means “no significant difference”; *P <* *.05. C, Representative Western blot results of microtubule protein for each group of PC12 cells. D‐E, Quantification of Western blot data from C, ***P <* *.001; **P <* *.01

To study the effect of AAV‐FGF1 in vivo, the AAV‐FGF1 virus was transferred to PC12 cells (50 pfu/cell). To simulate the microcirculation of SCI, we provoked PC12 cells with H_2_O_2_ (100 μmol/L) for 8 hours. We examined levels of ace‐tubulin, an indicator protein of the stabilized cytoskeleton, and Tau protein, a microtubule‐associated protein that plays a crucial role in microtubule assembly. Immunostaining of ace‐tubulin in PC12 cells was performed to evaluate the effect of FGF1 on cell morphology (Figure 5D). We observed a dramatic decrease in the axonal length of PC12 cells after exposure to H_2_O_2_ compared to the normal elongated or polarized cells (Figure 5A,B). However, cells of the H_2_O_2_ + AAV‐FGF1 group still showed polarized axons. As shown in Figure 5D,E, there were no obvious differences in the levels of ace‐tubulin and Tau between the groups without exposure to H_2_O_2_. And, FGF1 significantly ameliorated the loss of ace‐tubulin and increased expression of Tau (Figure 5A‐C), indicating that FGF1 effectively protected the cytoskeleton of neuron‐like cells against disintegration after extrinsic damage. Regulation of cytoskeleton structures is likely crucial for the regrowth of injured axons. Overall, PC12 cells were a reasonable model of neurons, showing significant protection of the cytoskeleton and a remarkable ability to repair injured axons by AAV‐FGF1.

### 3.5 FGF1 enhanced autophagy in vitro

**Figure 6:**
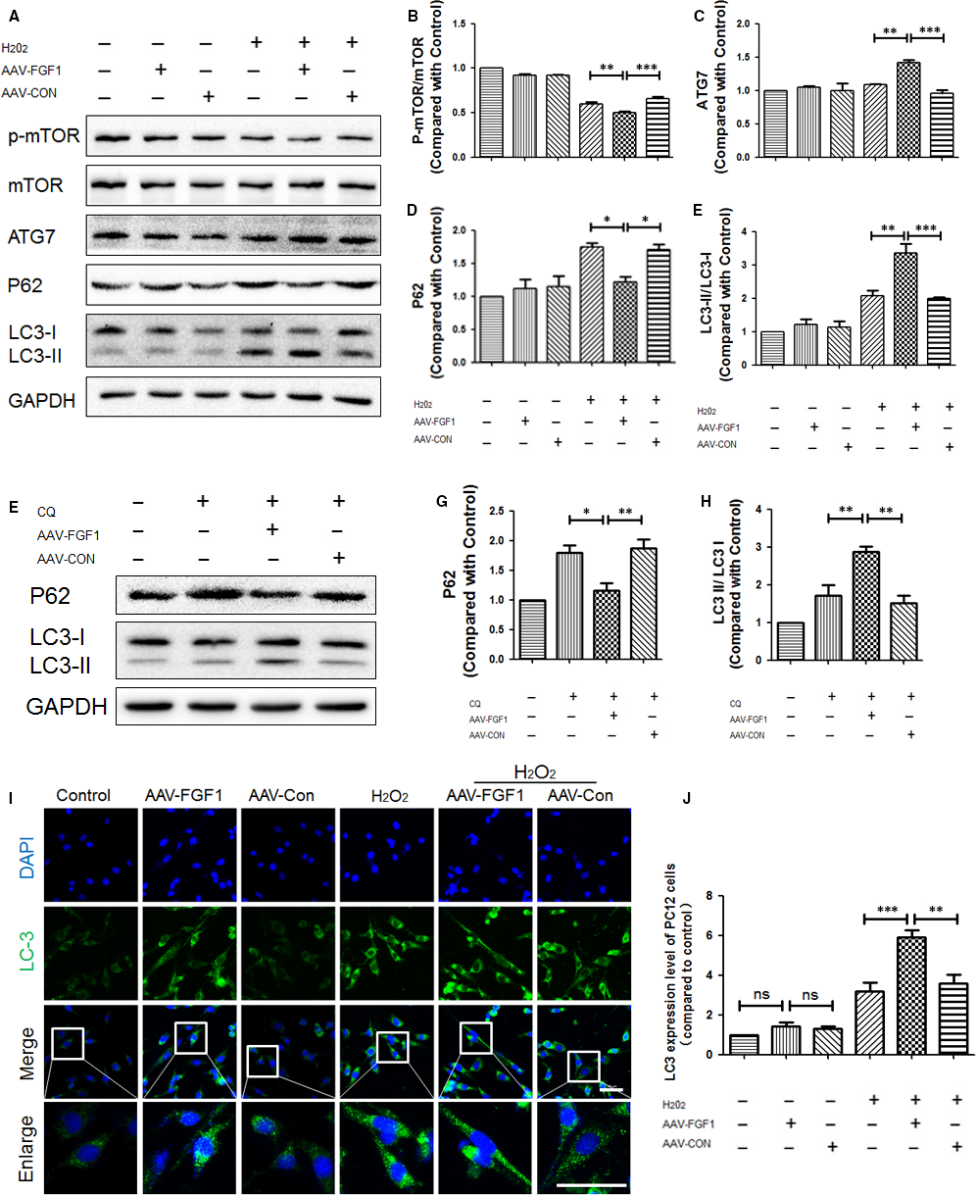
FGF1 enhanced autophagy in vitro. A, Representative Western blot results of autophagy‐associated protein for each group of PC12 cells. B‐E, Quantification of Western blot data from A, ***P <* *.001; **P <* *.01; *P <* *.05. F, Representative Western blot results of P62 and LC3 for each group of PC12 cells treated with chloroquine and AAV‐FGF1. G‐H, Quantification of Western blot data from F, **P <* *.01; *P <* *.05. I, Immunofluorescence images show LC3 (green) in PC12 cells subjected to H_2_O_2_ (100 μmol/L) and treated with AAV‐FGF1. Scale bar = 50 μm. J, Quantification of fluorescence (level of LC3) from I, ns means “no significant difference”; ***P <* *.001; **P <* *.01

In order to investigate the effect of FGF1 on autophagy in PC12 cells, the expression levels of autophagy‐associated proteins were measured after exposure to H_2_O_2_ and treatment with AAV‐FGF1. Interestingly, compared with the H_2_O_2_ group and the H_2_O_2_ + LV‐CON group, the phosphorylation of mTOR protein in the H_2_O_2_ + AAV‐FGF1 group was significantly decreased (Figure 6A,B). As shown in Figure 6A,C,D, the expression of ATG7 and LC3 II/I was apparently increased compared to the H_2_O_2_ and H_2_O_2_ + AAV‐CON groups. Furthermore, the expression of P62 of H_2_O_2_ + LV‐FGF1 groups was significantly decreased compared to the levels in the H_2_O_2_ and H_2_O_2_ + AAV‐CON groups. To examine the effect of FGF1 on autophagy flux in PC12 cells, CQ, a lysosomal inhibitor, was used to treat PC12 cells after transfection, and then, the expression levels of P62 and LC3 II/I were evaluated. As shown in Figure 6F‐H, the level of the P62 protein was obviously decreased in CQ + AAV‐FGF1 groups compared with the CQ and CQ + AAV‐CON groups. In addition, we performed immunostaining in PC12 cells to detect the expression level of LC3, and then, we observed that the degree of green fluorescence (LC3) was higher after treatment with AAV‐FGF1 than vehicle and AAV‐CON in a condition of H_2_O_2_ (Figure 6I‐J).

Taken together, AAV‐FGF1 increased autophagy in vitro. Combined with the previous data, we concluded that the up‐regulation of autophagy in vivo and in vitro by AAV‐FGF1 was probably associated with the effect of FGF1 on restoration of injured spinal cord or cells.

### 3.6 FGF1 induced PRDX1 expression to reduce the ROS

**Figure 7:**
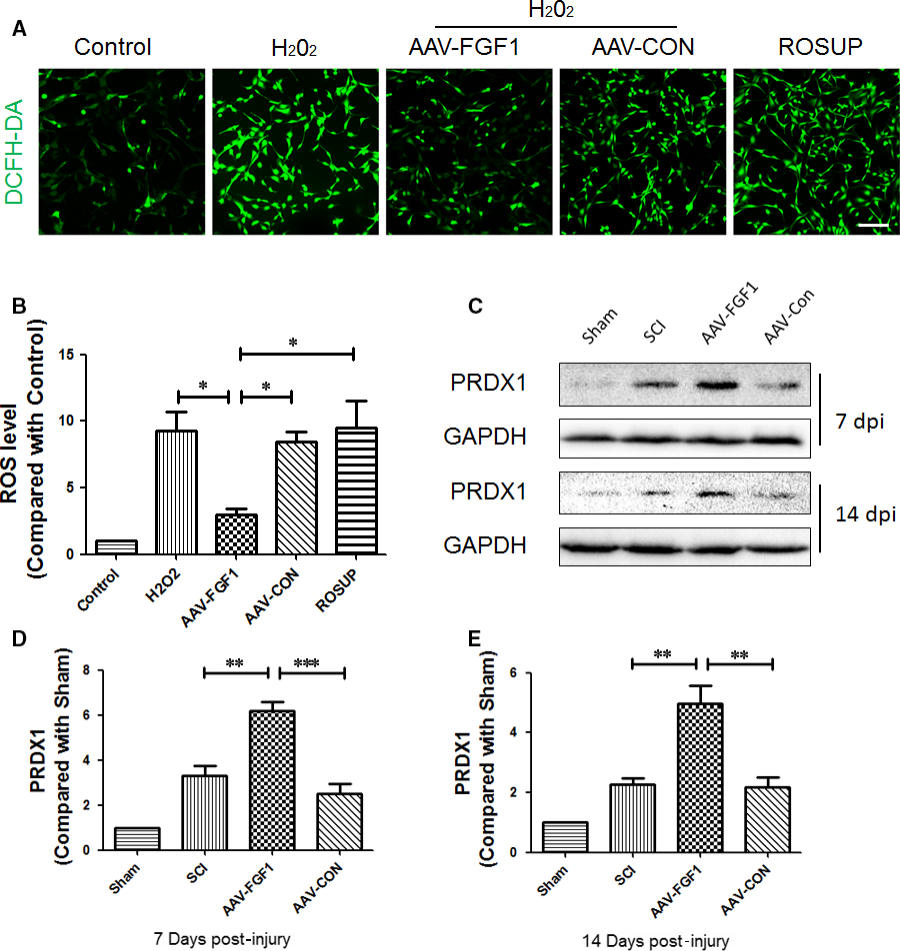
FGF1 increased PRDX1 and plays a protectional role in reactive oxygen species. A, The fluorescence images of the DCFH‐DA probe for hydrogen peroxide for each group of PC12 cells. Scale bar = 100 μm. B, Quantification of fluorescence density data from A, *P <* *.05. C, Representative Western blot result of autophagy‐associated proteins in each group at 7 and 14 dpi. D and E, Quantification of Western blot data from C, ***P <* *.001; **P <* *.01

ROS causes major detrimental effects during secondary injury and occurs together with many inflammatory conditions. Here, a specific probe for hydrogen peroxide, 2′, 7′‐dichlorodihydrofluorescein diacetate (DCFH‐DA) was used to evaluate the level of intracellular ROS. The fluorescence images showed a remarkably low density of fluorescence in the H_2_O_2_ + AAV‐FGF1 group compared with the H_2_O_2_ group, the H_2_O_2_ + LV‐CON group and the positive control group (ROSUP group) (Figure 7A,B), indicating that FGF1 has a significant effect on anti‐ROS. Next, we investigated PRDX1, an antioxidant protein, in injured spinal cord at 7 and 14 dpi (Figure 7C‐E). The result showed the up‐regulation of FGF1 mediated by AAV vector markedly enhanced the expression degree of PRDX1 at 7 and 14 dpi, an effect different from that seen in the SCI group and SCI + CON group. These data revealed a protectional role of FGF1 in SCI. Based on these results, PRDX1 and autophagy may counter the detrimental effect of ROS to promote recovery of SCI.

### 3.7 FGF1 regulates the level of autophagy via PRDX1 in vitro

**Figure 8:**
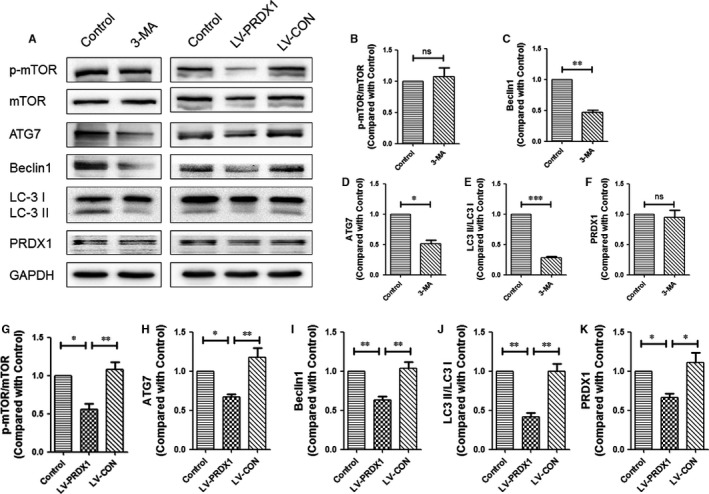
PRDX1 regulated the level of autophagy in vitro. A, Representative Western blots result of autophagy‐associated proteins and PRDX1 in each group of PC12 cells after using 3‐MA or LV‐PRDX1. B‐F, Quantification of Western blot data from A, ns means “no significant difference”; ***P <* *.001; **P <* *.01; *P <* *.05. G‐K, Quantification of Western blot data from A, **P <* *.01; *P <* *.05

**Figure 9:**
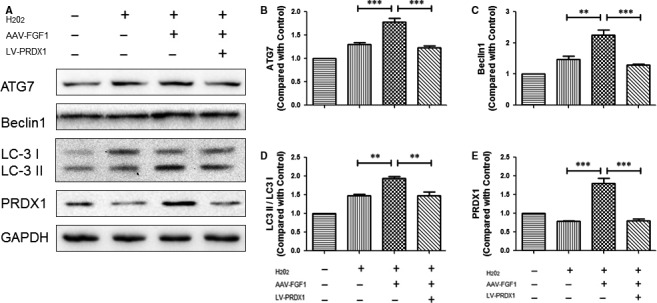
FGF1 regulates the level of autophagy via PRDX1 in vitro. A, Representative Western blot results of autophagy‐associated proteins and PRDX1 in each group of PC12 cells after using AAV‐FGF1 and LV‐PRDX1 sequentially. B‐E, Quantification of Western blot data from A, ***P <* *.001; **P <* *.01

To study the precise relationship between PRDX1 and autophagy, we detected the expression degree of autophagy and PRDX1 after treatment with 3‐methyladenine (3‐MA) and lentivirus‐PRDX1 (LV‐PRDX1, carrying siRNA against PRDX1) to inhibit autophagy and PRDX1, respectively. As shown in Figure 8A‐F, no significant difference was found in comparison with p‐mTOR/mTOR because 3‐MA inhibits Akt pathway, but not mTOR. The autophagy‐associated proteins, ATG7, Beclin1 and LC3 II/I, were markedly decreased in the 3‐MA group, suggesting that the intracellular autophagy was effectively inhibited by 3‐MA. Comparison of the PRDX1 levels revealed no significant difference between the control group and the 3‐MA group. In addition, as shown in Figure 8A,G‐K, after the expression of PRDX1 was apparently reduced by LV‐PRDX1, the level of p‐mTOR/mTOR was obviously decreased and the expression levels of ATG7, Beclin1 and LC3 II/I were significantly decreased. Considering these data, we concluded that the down‐regulation of PRDX1 may inhibit autophagy. In order to examine whether the up‐regulation of autophagy by FGF1 was via PRDX1 to affect the mTOR signal pathway, lentivirus‐PRDX1 (LV‐PRDX1) was used to interrupt the expression of PRDX1 after infecting AAV‐FGF1 in PC12 cells. The result showed that the autophagy‐associated proteins, ATG7, Beclin1 and LC3 II/I, were significantly decreased in the AAV‐FGF1 + LV‐PRDX1 group compared to the AAV‐FGF1 group after exposure to H_2_O_2_ (Figure 9A‐E). Consistent with the previous results, we concluded that the overexpression of FGF1 enhanced the expression of PRDX1 and then increased autophagy to counter ROS to allow recovery of SCI.

## 4 DISCUSSION

According to previous studies, the vital neuroprotective and neuroregenerative effects of FGF1 may be through the PI3K/Akt and MAPK/ERK pathways.^22^, ^23^, ^24^ However, there are still a few obstacles preventing clinical application of FGF1: (1) How to get the FGF1 protein across the BSCB; (2) How to target FGF1 to the lesion area of SCI; and (3) What precise molecular mechanisms or signal pathways are required during the treatment procedure. Recently, an increasing number of applications of gene transfer have been developed for SCI research, such as adenovirus and AAV. However, few studies have used an AAV vector to deliver FGF1 for treatment of the spinal cord after injury. Available data indicate that the AAV vector is safe for use with infected cells to release beneficial proteins to combat sublethal cell damage.^25^ Stimulated by this work, our present study shows that AAV‐FGF1 can be used as an efficient vector to sustain the intra‐expression of FGF1 at the lesion site of the spinal cord.

In this study, FGF1 was overexpressed at 14 days of post‐injury by infecting AAV into spinal cord tissue. The BBB scores and footprint analysis showed better motor behaviour in the SCI + AAV‐FGF1 group at 14 days of post‐injury. The H&E staining showed tissue restoration of FGF1 in the SCI + AAV‐FGF1 group. In addition, we evaluated the comprehensive therapeutic effect of FGF1 provided by the AAV‐FGF1 virus. Previous studies suggested that neuron loss,^26^ demyelination,^27^ axonal regeneration^28^ and microtubule stabilization^29^ were common indicators of the negative neurological effect of SCI. The release of FGF1 introduced by the AAV induced an increase in MBP, ace‐tubulin and GAP43, indicating that FGF1 participated in various aspects of SCI repair. We also performed LFB staining (reflecting remyelination), immunostaining for ace‐tubulin (reflecting axon regeneration) and staining for NeuN (reflecting neuron protection) to investigate the beneficial effects of FGF1. These results demonstrated that the release of FGF1 in the lesion area after delivery by the AAV could prevent the enlargement of the lesion area, demyelination and neuronal loss, and could promote axon regeneration. We concluded that it was more appropriate to use the AAV vector to deliver FGF1 rather than supply exogenous FGF1 directly because this approach can reduce the time and dosage of drug treatment, can induce a stable increase in FGF1 expression, and because localized overexpression can avoid the blocking effects of BSCB or metabolism by other organs. Thus, the results of this study may provide insight into the clinical application of FGF1.

According to previous studies, the molecular mechanism of FGF1 to repair damage of SCI is associated with PI3K/Akt, MAPK/ERK and endoplasmic reticulum stress.^30^ However, the exact mechanism was undefined. Our previous study showed that activation of autophagy was involved in the restoration of SCI.^20^, ^31^ Here, it was first to demonstrate that expression levels of autophagy‐associated proteins, such as ATG7, Beclin1 and LC3 II/I, were obviously increased in SCI at 7 days of post‐injury due to the release of FGF1 by cells infected by the AAV. Additionally, ROS is a crucial factor in secondary damage of SCI and impacts diverse processes, such as inflammation, apoptosis and autophagy. However, few studies have investigated the influence of autophagy on ROS. We detected a remarkable decrease in ROS in vitro and an increase in PRDX1, an important protein in anti‐ROS. These data indicate that FGF1 can regulate the level of ROS and autophagy during SCI, and suggest an interactional relationship.

Previous studies of PRDX1 have been concentrated in the field of cancer,^14^, ^32^ with few studies examining the role of PRDX1 in the field of CNS injury, such as SCI. In this present study, the results showed a special relationship between PRDX1 and autophagy. In PC12 cells, we detected autophagy‐associated proteins and PRDX1 after using 3‐MA and lentivirus‐PRDX1 to inhibit autophagy and PRDX1. Interestingly, our results showed that autophagy‐associated proteins, such as ATG7, Beclin1 and LC3 II/I, were significantly decreased after the inhibition of PRDX1, but no obvious alteration of PRDX1 was found after the inhibition of autophagy. Additionally, the results showed that the level of autophagy decreased after using lentivirus‐PRDX1 (interruption), AAV‐FGF1 (overexpression) and H_2_O_2_. Taken together, we conclude that PRDX1 regulation by FGF1 can impact autophagy and exert an efficient anti‐ROS effect.

To summarize, the results suggest that the beneficial effect of FGF1 induced is via an increase in PRDX1. The increase in PRDX1 stimulates the up‐regulation of autophagy and counters ROS. FGF1 facilitates neuroprotection, axon regeneration and remyelination. However, there are unanswered questions that need to be resolved in further studies. For example, the exact target and the route for PRDX1 action on the autophagy pathway remain elusive. To conclude, our results identify an effective delivery strategy for FGF1 in SCI therapy and explored the precise mechanism of autophagy and ROS after SCI. This work provides the theoretical basis of therapeutic effect of FGF1 in acute and chronic patients with spinal cord injury.

## CONFLICT OF INTEREST

The authors declare that they have no conflicts of interest.
